# Gastrointestinal perforation associated with novel antineoplastic agents: A real-world study based on the FDA Adverse Event Reporting System

**DOI:** 10.3389/jpps.2023.11235

**Published:** 2023-02-15

**Authors:** Zicheng Yu, Haibin Zhu, Hongjun Chen, Lifei Zhu, Xiaolan Liao

**Affiliations:** Department of Pharmacy, Yangpu Hospital, School of Medicine, Tongji University, Shanghai, China

**Keywords:** gastrointestinal perforation, novel antineoplastic agents, adverse event, real-world study, pharmacovigilance

## Abstract

**Purpose:** Gastrointestinal perforation (GIP) is a fatal adverse event (AE). The AE of GIP induced by novel antineoplastic agents has attracted attention recently. We aimed to explore the AE signals of GIP related to novel antineoplastic agents comprehensively based on the FDA Adverse Event Reporting System (FAERS).

**Methods:** The FAERS database containing 71 quarters of records was used for analysis. Reporting odds ratio (ROR), information component (IC), and empirical Bayesian geometric mean (EBGM) were utilized to evaluate the signals of GIP associated with novel antineoplastic drugs. Standardization of drug names was by employing MedEx-UIMA software and Python. Data analysis and visualization were performed using MySQL Workbench and R software.

**Results:** After cleaning and handling the data, 5226 GIP cases were identified that were associated with new antineoplastic medications, where these agents were the main suspected contributors. A total of 37 novel antineoplastic drugs were detected with signals of GIP for ROR and IC. Only 22 drugs showed statistically significant signals for EBGM. We found the GIP signals of 22 novel antineoplastic drugs overlapped for the 3 indicators, including anti-vascular endothelial growth factor/vascular endothelial growth factor receptor, anti-endothelial growth factor receptor, immune checkpoint inhibitors, and so on.

**Conclusion:** The potential risk of GIP associated with several novel antineoplastic agents was identified through data mining, which provided valuable information on the safety risks associated with GIP among these drugs. The potential threat of GIP should be recognized and managed properly when using these novel antineoplastic agents.

## Introduction

Gastrointestinal perforation (GIP) is a severe and relatively uncommon adverse event (AE), which is potentially mortal. Clinical attention has been drawn to GIP related to drugs owing to its serious outcome. The GIP may result from the use of drugs such as non-steroidal anti-inflammatory drugs, anticoagulants, corticosteroids, some antineoplastic agents, and some other drugs (1–4).

Developments and innovations in pharmaceutical industries have given rise to numerous new and effective anti-cancer treatments, such as small-molecule targeted drugs and monoclonal antibodies. Antibody-drug conjugates, programmed cell death protein 1 (PD-1)/programmed death ligand-1 (PD-L1), chimeric antigen receptor-T-cells (CRA-T), and so on that are rapidly emerging as a boon for oncology patients (5, 6). The gastrointestinal AE induced by the novel antineoplastic agents has been mentioned commonly, however, the AE of GIP associated with these new antineoplastic drugs is rarely reported. FDA added a black box and recommended permanent discontinuation of bevacizumab in patients with GIP (7). The inhibitors of vascular endothelial growth factor (VEGF) and its receptor (VEGFR) were potentially associated with GIP (8, 9). With more and more novel antineoplastic agents are available, it is of great clinical practical significance to systematically study the relationship between novel antineoplastic agents and GIP. Unfortunately, no large-scale and comprehensive research has confirmed this association yet.

The vast database of AE reports established by the U.S. FDA provides abundant real-world data on the safety of drugs of clinical applications of prescriptions (10). Most of all, it is a database that’s free and accessible to the public (11). Data mining is recognized as an excellent method for early detection of drug safety signals, which can be applied to predict and understand the safety of drugs (12). Pharmacovigilance research based on the combination of data mining methods with the FDA Adverse Event Reporting System (FAERS) database has become progressively prevalent in recent years. In our study, we used the FAERS database to provide reference evidence by calculating the AE signals with a view to a better understanding of the association between GIP and new antineoplastic agents for safe clinical use.

## Methods

### Data source

FAERS updates AE data quarterly. We retrieved all the data from the official website (11). Two kinds of data formats (ASCII and XML) are available, both of which are common and convenient to manage. The ASCII files for quarter 1 of 2004 (2004Q1) to quarter 3 of 2021 (2021Q3) were fetched. Each quarter file contains seven subfiles, providing information separately, as shown in Table 1.

**TABLE 1:** The contents of data in the seven subfiles provided by FAERS.

Filename	Contents of data
DEMO	Patient demographic and administrative information
DRUG	Drug information
INDI	Indications for drug administration
OUTC	Patient outcomes
REAC	Adverse events
RPSR	Report sources
THER	Therapy start dates and end dates for reported drugs

### Discrimination of objective drugs and AE

There are two fields in the DRUG files that relate to drug names, including the “drugname” and the “prod_ai.” Given that FAERS is a spontaneous reporting system (SRS), drug names may be documented as generic names, chemical structure names, trade names, synonyms, code names, abbreviations, and even incorrect names (13). Therefore, before data mining, all drug name records were standardized in the DEMO files as generic names. Firstly, the “drugname” and “prod_ai” fields were mapped to the specific RxNorm concepts, which contain a single active ingredient by using MedEx-UIMA software (MedEx-UIMA 1.3.8, Vanderbilt University, United States) (14). Secondly, we merged the fields containing compound ingredients after the first step of processing by using python 3.8 (Python Software Foundation, Wilmington, United States), and the pharmacist manually standardized the drug names. Finally, the data cleaning of the DRUG table was completed by screening and checking once again, by using the DrugBank database (15). The target drugs are novel antineoplastic agents, including but not limited to the following types of drugs: small-molecule targeted drugs as well as monoclonal antibodies. To minimize the probability of false positives, we selected only “role_cod” fields that play the role of “primary suspect (PS).”

The “pt” field in the REAC table is the name of AE written in the Medical Dictionary for Regulatory Activities (MedDRA) and represented as the “Preferred Terms” (PTs). The Standardised MedDRA (MedDRA^®^ trademark is registered by ICH, version 24.0) queries (SMQs) was employed to access PTs related to GIP (16). A total of 24 PTs were identified as target AE terms after referring to books and literature, as described below: “Duodenal perforation,” “Duodenal ulcer perforation,” “Duodenal ulcer perforation, obstructive,” “Gastric perforation,” “Gastric ulcer perforation, obstructive,” “Gastrointestinal perforation,” “Ileal perforation,” “Ileal ulcer perforation,” “Intestinal perforation,” “Jejunal perforation,” “Jejunal ulcer perforation,” “Large intestine perforation,” “Oesophageal perforation,” “Peptic ulcer perforation,” “Rectal perforation,” “Small intestinal perforation,” “Oesophageal ulcer perforation,” “Large intestinal ulcer perforation,” “Small intestinal ulcer perforation,” “Intestinal ulcer perforation,” “Diverticular perforation,” “Gastrointestinal ulcer perforation,” “Upper gastrointestinal perforation,” “Lower gastrointestinal perforation.”

### Statistical analysis

We mainly used three files, DEMO, DRUG, and REAC. According to FDA’s recommendations, data cleaning was first performed for the DEMO table. Above all, duplicate records were excluded. If the CASEID (the number used to identify FAERS cases) is the same, the latest FDA_DT (the date the FDA received the case) is picked. If the CASEID and FDA_DT are the same, the higher PRIMARYID (the unique value used to identify the reports) is selected.

Descriptive analysis was performed to describe information about the case, including gender, age, occupation of the reporter, and year of reporting. The following three indicators, including reporting odds ratio (ROR), information component (IC), and empirical Bayesian geometric mean (EBGM) were applied to measure the signals of GIP of the target drug (17). The 95% two-sided confidence interval (CI) was calculated. The ROR is based on frequentist and it is extensively utilized because it’s simple to calculate and understand (18, 19). ROR05 (the lower limit of the 95% two-sided CI of the ROR) > 1 and the number of cases≥2 is considered with statistically significant signal (19–21). The IC is calculated by the Bayesian confidence propagation neural network (BCPNN) algorithm that was developed by the Uppsala Monitoring Center (UMC) (22, 23). The IC025 (the lower limit of the 95% two-sided CI of the IC) > 0 is regarded as with statistically significant signal (22, 24). The EBGM is based on the multi-item gamma Poisson shrinker (MGPS) algorithm, EBGM05 (the lower limit of the 95% two-sided CI of the EBGM) > 2, and the number of cases ≥0 is considered with statistically significant signal (25, 26). BCPNN and MGPS are both based on Bayesian methods, which tend to be more conservative in simulation studies than other methods and detect less signal in real data examples. As a result, BCPNN and MGPS have lower sensitivity and better specificity (18, 27). The calculation formulas and judging criteria for the three indicators are summarized in Supplementary Tables S1, S2.

## Results

**FIGURE 1:**
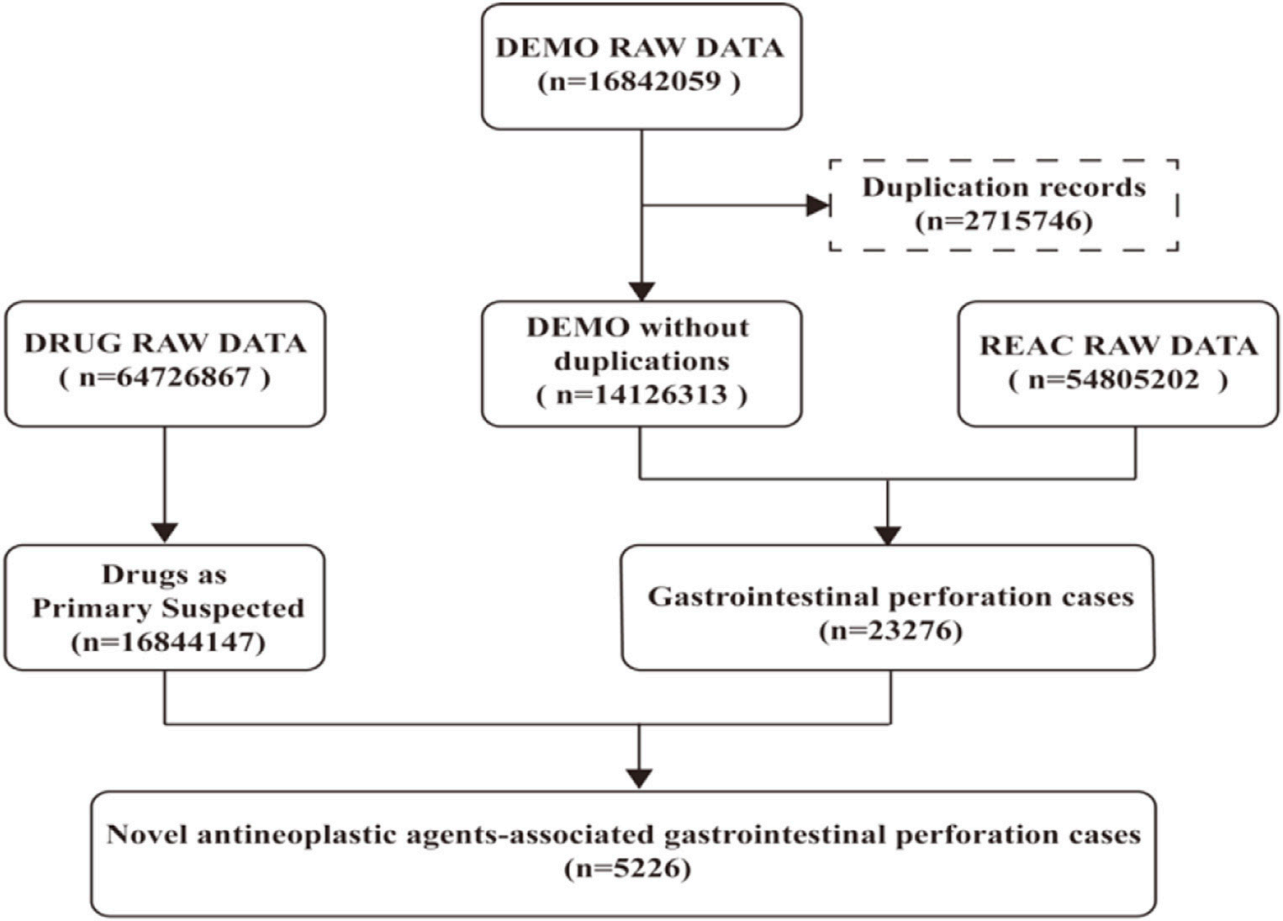
Flowchart of identifying GIP cases associated with novel antineoplastic drugs.

The DEMO files in the FAERS database documented 16842059 reports in the past 18 years, and 14126313 records were included in our study after deduplicating by following the FDA recommendations. 23276 cases were identified as GIP, of which 5,226 cases were judged being associated with the new antineoplastic medications (Figure 1). The clinical baseline data of the 5,226 cases was presented in Table 2. The sex percentage of reported cases were males (29.90%) verse females (26.90%). More than half of the patients were over 45 years old (63.61%).

**TABLE 2:** Summary of patient characteristics with the AE of GIP related to novel antineoplastic agents.

Class	Cases (n)	Percent (%)
Sex		
Female	1408	26.90
Male	1565	29.90
Unclear or Missing	2,253	43.10
Age		
0–18	21	0.40
19–45	285	5.45
46–65	1586	30.35
65–100	1738	33.26
unclear or Missing	1596	30.54
Reporter		
Consumer	844	16.15
Physician	2,792	53.43
Pharmacist	256	4.90
Other health-professional	1155	22.10
Unclear or Missing	179	3.43
Report Years		
2022	508	9.72
2021	525	10.05
2020	531	10.16
2019	443	8.48
2017	563	10.77
2018	360	6.89
2004–2016	2,291	43.84
Unclear or Missing	5	0.09

**FIGURE 2:**
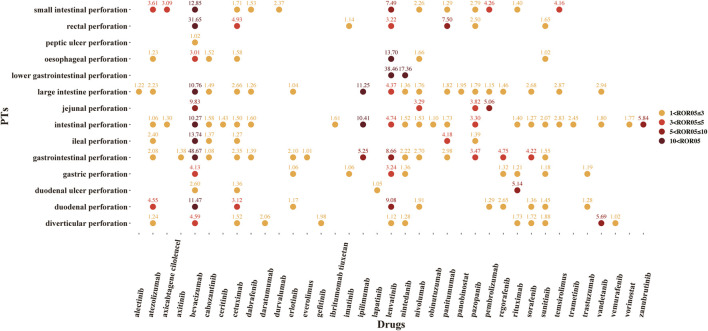
The signals of reporting odds ratio (ROR) of novel antineoplastic agents in detailed GIP AE PTs, preferred terms; ROR05, the lower end of the 95% confidence interval of ROR (ROR05 > 1 was regarded as the statistically significant signals).

**FIGURE 3:**
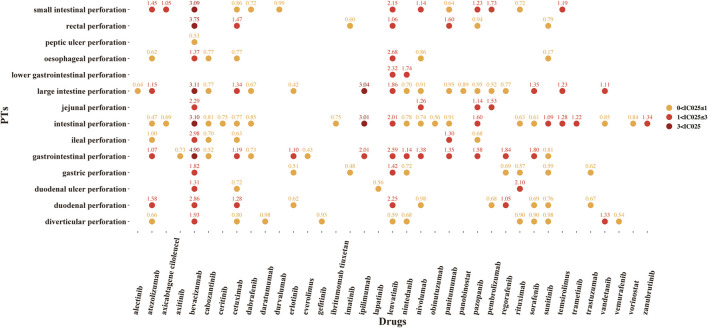
The signals of information component (IC) of novel antineoplastic drugs in detailed GIP AE PTs, preferred terms; IC025, the lower end of the 95% confidence interval of IC (IC025 > 0 was regarded as the statistically significant signals).

**FIGURE 4:**
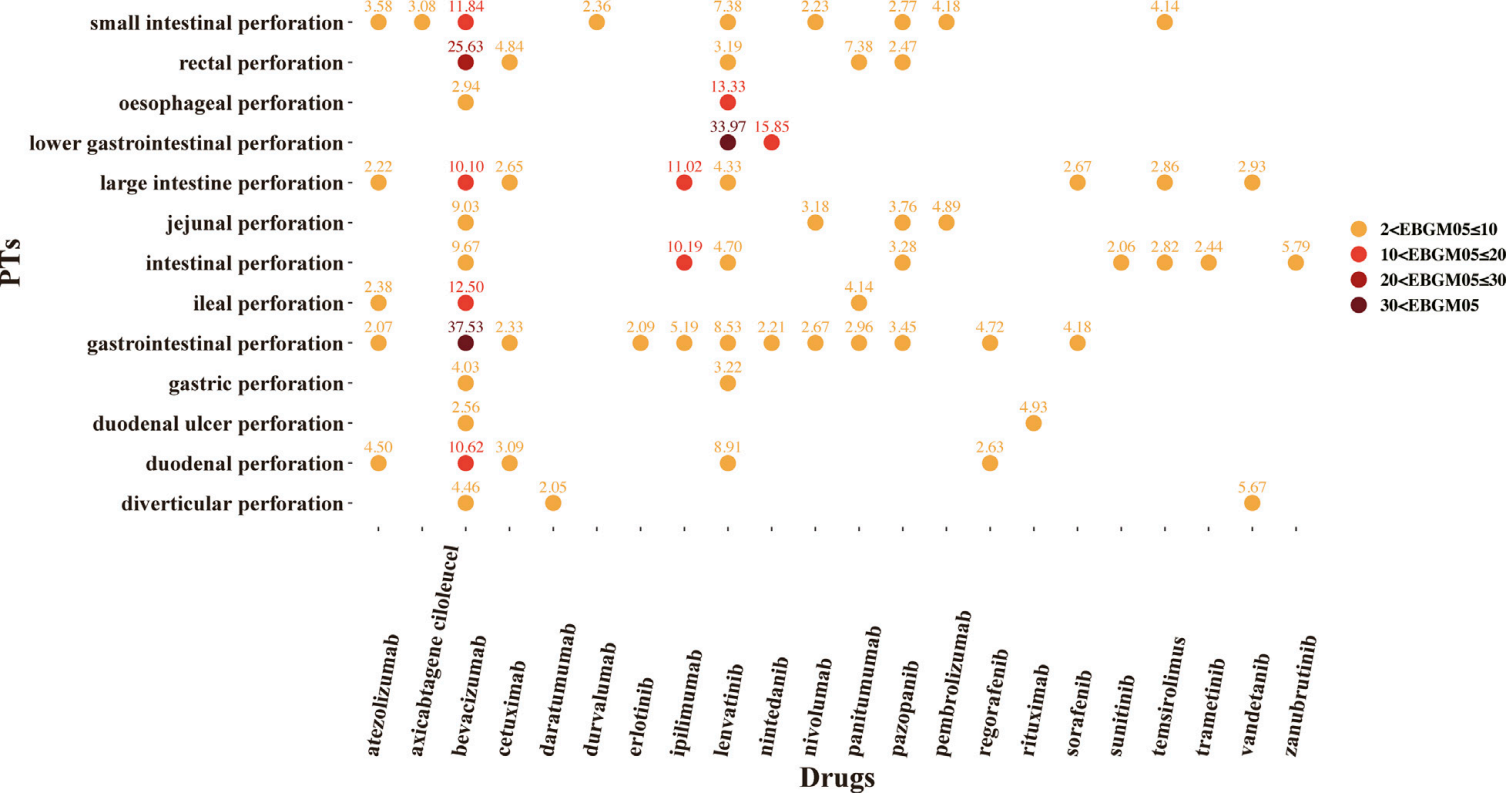
The signals of empirical Bayesian geometric mean (EBGM) of novel antineoplastic drugs in detailed GIP AE PTs, preferred terms; EBGM05, the lower end of the 95% confidence interval of EBGM (EBGM05 > 2 was regarded as the statistically significant signals).

A total of 37 novel antitumor drugs were detected with signals of GIP in terms of ROR and IC indexes (Figures 2, 3). Bevacizumab was identified with 13 signals that ranked the first, and the PT with the strongest signal was “gastrointestinal perforation” (ROR05 = 53.18, IC025 = 4.90). Both cetuximab and lenvatinib ranked the second concurrently, each with 10 statistically significant signals. For cetuximab, the strongest signal was “rectal perforation” (ROR05 = 4.90, IC025 = 1.47). The strongest signal of PT was “lower gastrointestinal perforation” for lenvatinib (ROR05 = 38.46, IC025 = 2.32). Atezolizumab was found with 8 signals, and the strongest signal of PT was “duodenal perforation” (ROR05 = 4.55, IC025 = 1. 58). In contrast, only 22 drugs presented statistically significant signals based on the EBGM (Figure 4). The detailed results were shown in Supplementary Table S3.

There were 37 novel antitumor drugs being detected with signals of GIP based on ROR and IC indexes. Totally, for ROR, IC, and EBGM indicators, we found that there were 22 novel antineoplastic agents associated with overlapping GIP-related signals that including anti-VEGF/VEGFR, anti-endothelial growth factor receptor (EGFR), PD-1/PD-L1, and so on. The targets of the 22 drugs are summarized in Table 3.

**TABLE 3:** Association of GIP signals with novel antineoplastic agents for the overlapped signals of the 3 indicators.

Drugs	Primary target
TKIs	
erlotinib	EGFR
lenvatinib	VEGFR
nintedanib	VEGFR
pazopanib	VEGFR
regorafenib	VEGFR
sorafenib	VEGFR
sunitinib	VEGFR
trametinib	MEK
vandetanib	VEGFR
zanubrutinib	Bruton’s tyrosine kinase
Monoclonal Antibodies	
ICIs	
atezolizumab	PD-L1
durvalumab	PD-L1
nivolumab	PD-1
pembrolizumab	PD-1
ipilimumab	CTLA-4
Others Monoclonal Antibodies	
bevacizumab	VEGF
cetuximab	EGFR
daratumumab	CD 38
panitumumab	EGFR
rituximab	CD 20
Others	
axicabtagene ciloleucel	CD19-expressing cells
temsirolimus	mTOR

TKIs, tyrosine kinase inhibitors; EGFR, epidermal growth factor receptors; VEGF, vascular endothelial growth factor; VEGFR, vascular endothelial growth factor receptor; MEK (also known as MAP2K or MAPKK), mitogen-activated protein kinase kinase; ICIs, immune checkpoint inhibitors; PD-L1, programmed death ligand-1; PD-1, programmed cell death protein-1; CTLA-4, Cytotoxic T-lymphocyte-associated Protein-4; CD, cluster of differentiation; CAR-T, chimeric antigen receptor T-cell; mTOR, mammalian target of rapamycin.

## Discussion

The availability of novel antineoplastic drugs, especially for molecularly targeted drugs and tumor immune agents have dramatically changed the status of tumor treatment, and more and more patients benefit from these new treatments. However, these novel antineoplastic drugs have the risk of gastrointestinal perforation, a rare but fatal adverse event.

Bevacizumab was found to have the most potential to increase the risk of GIP, Wichelmann et al. performed a descriptive study of GIP with bevacizumab based on the FAERS database (9). A meta-analysis showed that there was no statistical difference in the risk of GIP between VEGFR-tyrosine kinase inhibitors (TKIs) and control groups (28). However, cases of GIP caused by anti-VEGF/VEGFR agents have been continuously reported in recent years (29–31). Wang Z et al. found that ramucirumab, a novel anticancer drug that belongs to the class of anti-VEGF agents, is associated with a significant increase in the risk of GIP (32). No comprehensive study has been conducted for comparative evaluation of the relationship of GIP associated with VEGF/VEGFR inhibitors. Data mining was applied to comprehensively estimate the GIP pharmacovigilance signal associated with the use of VEGF/VEGFR inhibitors. We found that VEGF/VEGFR inhibitors were associated with GIP, especially for bevacizumab, lenvatinib, and vandetanib.

The exact pathological mechanism by which VEGFR inhibitors cause perforation of the gastrointestinal tract remains unclear. However, the following inferences may be rational. Firstly, VEGF inhibition is thought to interfere with platelet-endothelial cell interactions, which may lead to loss of gastrointestinal vascular integrity and gastrointestinal submucosal inflammation (33). Secondly, these medications may reduce the blood delivery to the intestinal wall from normal blood vessels, which can lead to ischemia of the gastrointestinal mucosa (34). Finally, patients are immunocompromised on account of chemotherapy, which may cause dysbiosis of the intestinal flora and increase the risk of GIP (35).

Anti-EGFR drugs seems to be associated with GIP in our present study, including erlotinib, cetuximab and panitumumab. To date, there are fewer reports of anti-EGFR agents associated with GIP. Gass-Jégu F. et al. reported two cases of GIP after taking erlotinib treatment (36). Anti-EGFR drugs may downregulate VEGF expression, which leads to ischemia in gastrointestinal tract tissues (34, 37). However, the official drug label of cetuximab and panitumumab didn’t mention GIP. The relationship of anti-EGFR with GIP needs to be confirmed by further studies with different analysis tools.

In recent years, immune checkpoint inhibitors (ICIs) are the focus of research in the field of tumor immunotherapy. Inhibitors of PD-1/PD-L1 and cytotoxic T-lymphocyte-associated protein-4 (CTLA-4) targets have demonstrated significant therapeutic benefits in clinical applications (38–41). Gastrointestinal AEs are the most common immune-related AEs (irAEs) with ICIs and the essential reason for the discontinuation of ICIs (42–44). The GIP signals associated with atezolizumab, durvalumab, nivolumab, pembrolizumab, and ipilimumab were detected by all three indicators. For official drug label, nivolumab reported with GIP, pembrolizumab and ipilimumab reported with GIP only in combination, atezolizumab, and durvalumab were not reported in the instructions. Although our study differs from the manual report, it is undeniable that ICIs cause a high proportion of immune enterocolitis, which has the potential risk of causing GIP (45, 46). GIP signals associated with ICIs were detected by using the VigiBase database that confirmed our research (47). More and more studies reported cases of GIP after taking PD-1 (48, 49). Therefore, these signals need to be paid more attention. Mechanisms of GIP toxicity associated with ICIs remain to be clarified, however, it is considered that T cells, antibodies and cytokines contribute to the development of irAEs (50–52).

The TKIs of trametinib and zanubrutinib were detected with the GIP signals. Trametinib is one of the mitogen-activated protein kinase kinase (MEK, also known as MAP2K or MAPKK) inhibitors. Mourad N. et al. found that patients suffered GIP with the treatment of MEK inhibitors (53). The monoclonal antibodies of rituximab also showed the GIP signals. The GIP of rituximab therapy is rare but there, and several case reports indicated a possible association between rituximab and GIP (54–56). Although rare, rituximab might be associated with colitis that can be severe enough to cause colon perforation (57). Noteworthy, axicabtagene ciloleucel, one of the CAR-T therapies, was detected with the potential GIP signals in our study. A post-marketing surveillance study showed the new potential GIP signal for axicabtagene ciloleucel (58). A clinical study of the late effects of CAR-T cells reported that about 1.8% of patients died from duodenal ulcer and gut perforation (59). Because CAR-T immunotherapy is a novel antineoplastic treatment, its relationship with GIP still needs to be validated with a larger amount of post-marketing clinical data. Nevertheless, it is of great significance to learn about their potential and fatal AE.

One point to note is that no GIP was reported in the official drug label for zanubrutinib, cetuximab, panitumumab, daratumumab, and axicabtagene ciloleucel, but we detected relevant signals. It may be caused by unknown confounding factors or the limitations of the search and analytical conduct of the study. Furthermore, the signals detected in our study could be suggestive of potential risks, and whether there is an association between GIP and these drugs needs to be evaluated and interpreted by combining official drug labels, expert opinion, clinical data, and other relevant factors. Therefore, caution is needed to interpret our results showing the association of these drugs with GIP. However, GIP is a very serious AE, and these signals should still raise our alert.

The FAERS database provides a tremendous amount of information on AE, but unavoidably, limitations remain in our study. Firstly, it is inevitable that the clinical information might be missing or unknown because the FAERS is based on the SRS. Secondly, the total number of patients using these drugs cannot be accessed, so the exact incidence is extremely difficult to be calculated. Thirdly, to avoid false positives results, we considered only ‘PS’ agents and did not take into account concomitant drugs. The relevant drug interaction studies will be conducted in the future. Last but not least, due to the presence of confounding factors, the incidence and risk of GIP associated with those agents need to be studied and evaluated in prospective studies.

## Conclusion

The potential risk of GIP associated with several novel antineoplastic agents, including anti-VEGF/VEGFR agents, anti-EGFR drugs, ICIs, etc., was comprehensively investigated by data mining. Our present study would provide valuable information on the different safety risks associated with GIP among these drugs. GIP could lead to fatal consequences, therefore, the potential threat of GIP should be kept in mind during the use of these novel antineoplastic agents. And the potential risk of GIP needs to be recognized and managed properly.

## Data Availability

The original contributions presented in the study are included in the article/Supplementary Material, further inquiries can be directed to the corresponding author.
