# Supplementary material for: Gastrointestinal perforation associated with novel antineoplastic agents: A real-world study based on the FDA Adverse Event Reporting System
Source: J Pharm Pharm Sci. 2023 Feb 15;26:11235. doi: 10.3389/jpps.2023.11235 (PMC9990630; doi:10.3389/jpps.2023.11235)
Supplement: Supplementary file 1 [file DataSheet1.docx]

**Table S1.** Two-by-two contingency table for analysis.

| Drugs | Hypertension cases | All other adverse event cases | Total |
| --- | --- | --- | --- |
| VEGFR-TKIs | a | b | a+b |
| All other drugs | c | d | c+d |
| Total | a+c | b+d | a+b+c+d |

**Table S2.** Summary of major algorithms used for signal detection.

| Algorithms | Indicator | Equation | Criteria |
| --- | --- | --- | --- |
| ROR | ROR | ROR=ad/c/b | ROR05>1, N≥2 |
|  |  | 95%CI=e^ln(ROR)±1.96(1/a+1/b+1/c+1/d)^0.5^ |  |
| BCPNN | IC | IC=log_2_[a(a+b+c+d)]/[(a+c)(a+b)] | IC025>0 |
|  |  | 95%CI=e^ln(IC)±1.96(1/a+1/b+1/c+1/d)^0.5^ |  |
| MGPS | EBGM | EBGM=a(a+b+c+d)/(a+c)/(a+b) | EBGM05>2, N≥0 |
|  |  | 95%CI=e^ln(EBGM)±1.96(1/a+1/b+1/c+1/d)^0.5^ |  |

N, number of adverse event reports; CI, confidence interval; ROR, reporting odds ratio; ROR05, the lower limit of the 95% two-sided CI of the ROR; N, the number of co-occurrences; PRR, proportional reporting ratio; χ^2^, chi-squared; BCPNN, Bayesian confidence propagation neural network; IC, information component; IC025, the lower limit of the 95% two-sided CI of the IC; MGPS, multi-item gamma Poisson shrinker; EBGM, empirical Bayesian geometric mean; EBGM05, the lower 95% two-sided CI of EBGM.

**Table S3.** Summary of gastrointestinal perforation signals associated with new antineoplastic agents of the 3 indicators.

| Drugs | PT | N | ROR (95% CI) | IC (95% CI) | EBGM (95% CI) |
| --- | --- | --- | --- | --- | --- |
| atezolizumab | small intestinal perforation | 9 | 6.96 (3.61,13.43) | 2.79 (1.45,5.37) | 6.90 (3.58,13.31) |
| atezolizumab | oesophageal perforation | 3 | 3.84 (1.23,11.94) | 1.93 (0.62,6.02) | 3.82 (1.23,11.89) |
| atezolizumab | large intestine perforation | 22 | 3.39 (2.23,5.15) | 1.75 (1.15,2.67) | 3.37 (2.22,5.13) |
| atezolizumab | intestinal perforation | 18 | 1.69 (1.06,2.68) | 0.75 (0.47,1.2) | 1.69 (1.06,2.68) |
| atezolizumab | ileal perforation | 4 | 6.41 (2.4,17.17) | 2.67 (1,7.15) | 6.36 (2.38,17.03) |
| atezolizumab | gastrointestinal perforation | 14 | 3.52 (2.08,5.94) | 1.81 (1.07,3.06) | 3.5 (2.07,5.92) |
| atezolizumab | duodenal perforation | 8 | 9.14 (4.55,18.36) | 3.17 (1.58,6.38) | 9.03 (4.5,18.14) |
| atezolizumab | diverticular perforation | 6 | 2.77 (1.24,6.17) | 1.46 (0.66,3.27) | 2.76 (1.24,6.15) |
| axicabtagene ciloleucel | small intestinal perforation | 3 | 9.6 (3.09,29.82) | 3.26 (1.05,10.12) | 9.56 (3.08,29.72) |
| axicabtagene ciloleucel | intestinal perforation | 7 | 2.73 (1.3,5.73) | 1.45 (0.69,3.04) | 2.73 (1.3,5.73) |
| bevacizumab | small intestinal perforation | 76 | 16.26 (12.85,20.56) | 3.90 (3.09,4.94) | 14.98 (11.84,18.95) |
| bevacizumab | rectal perforation | 48 | 43.38 (31.65,59.46) | 5.13 (3.75,7.04) | 35.12 (25.63,48.14) |
| bevacizumab | peptic ulcer perforation | 3 | 3.19 (1.02,9.99) | 1.66 (0.53,5.19) | 3.15 (1.01,9.87) |
| bevacizumab | oesophageal perforation | 15 | 5.02 (3.01,8.39) | 2.3 (1.37,3.84) | 4.91 (2.94,8.20) |
| bevacizumab | large intestine perforation | 289 | 12.12 (10.76,13.66) | 3.51 (3.11,3.95) | 11.39 (10.10,12.83) |
| bevacizumab | jejunal perforation | 14 | 17.01 (9.83,29.42) | 3.97 (2.29,6.86) | 15.62 (9.03,27.02) |
| bevacizumab | intestinal perforation | 442 | 11.31 (10.27,12.45) | 3.41 (3.10,3.76) | 10.65 (9.67,11.72) |
| bevacizumab | ileal perforation | 42 | 18.88 (13.74,25.95) | 4.1 (2.98,5.64) | 17.17 (12.50,23.60) |
| bevacizumab | gastrointestinal perforation | 638 | 53.18 (48.67,58.10) | 5.36 (4.9,5.85) | 41.01 (37.53,44.80) |
| bevacizumab | gastric perforation | 51 | 5.45 (4.13,7.21) | 2.41 (1.82,3.19) | 5.32 (4.03,7.03) |
| bevacizumab | duodenal ulcer perforation | 34 | 3.65 (2.60,5.12) | 1.85 (1.31,2.59) | 3.59 (2.56,5.05) |
| bevacizumab | duodenal perforation | 49 | 15.35 (11.47,20.56) | 3.83 (2.86,5.13) | 14.22 (10.62,19.03) |
| bevacizumab | diverticular perforation | 50 | 6.08 (4.59,8.06) | 2.56 (1.93,3.40) | 5.91 (4.46,7.84) |
| cetuximab | small intestinal perforation | 6 | 3.81 (1.71,8.51) | 1.92 (0.86,4.29) | 3.79 (1.70,8.46) |
| cetuximab | rectal perforation | 5 | 11.95 (4.93,28.98) | 3.55 (1.47,8.61) | 11.73 (4.84,28.44) |
| cetuximab | oesophageal perforation | 4 | 4.22 (1.58,11.29) | 2.07 (0.77,5.54) | 4.2 (1.57,11.23) |
| cetuximab | large intestine perforation | 30 | 3.81 (2.66,5.46) | 1.92 (1.34,2.75) | 3.79 (2.65,5.43) |
| cetuximab | intestinal perforation | 28 | 2.17 (1.50,3.14) | 1.11 (0.77,1.61) | 2.16 (1.49,3.13) |
| cetuximab | ileal perforation | 3 | 3.95 (1.27,12.31) | 1.98 (0.63,6.15) | 3.93 (1.26,12.24) |
| cetuximab | gastrointestinal perforation | 18 | 3.73 (2.35,5.93) | 1.89 (1.19,3.01) | 3.71 (2.33,5.90) |
| cetuximab | duodenal ulcer perforation | 8 | 2.72 (1.36,5.45) | 1.44 (0.72,2.88) | 2.71 (1.35,5.43) |
| cetuximab | duodenal perforation | 7 | 6.58 (3.12,13.85) | 2.70 (1.28,5.69) | 6.51 (3.09,13.72) |
| cetuximab | diverticular perforation | 8 | 3.04 (1.52,6.10) | 1.60 (0.8,3.21) | 3.03 (1.51,6.07) |
| daratumumab | diverticular perforation | 6 | 4.59 (2.06,10.23) | 2.19 (0.98,4.89) | 4.57 (2.05,10.19) |
| durvalumab | small intestinal perforation | 4 | 6.33 (2.37,16.89) | 2.66 (0.99,7.09) | 6.3 (2.36,16.83) |
| erlotinib | large intestine perforation | 26 | 1.53 (1.04,2.26) | 0.61 (0.42,0.90) | 1.53 (1.04,2.25) |
| erlotinib | gastrointestinal perforation | 31 | 3.00 (2.10,4.27) | 1.57 (1.10,2.24) | 2.98 (2.09,4.24) |
| erlotinib | gastric perforation | 12 | 1.88 (1.06,3.31) | 0.9 (0.51,1.59) | 1.87 (1.06,3.30) |
| erlotinib | duodenal perforation | 6 | 2.62 (1.17,5.85) | 1.38 (0.62,3.08) | 2.6 (1.16,5.81) |
| ipilimumab | large intestine perforation | 83 | 13.99 (11.25,17.39) | 3.78 (3.04,4.69) | 13.7 (11.02,17.02) |
| ipilimumab | intestinal perforation | 121 | 12.47 (10.41,14.93) | 3.61 (3.01,4.32) | 12.21 (10.19,14.62) |
| ipilimumab | gastrointestinal perforation | 28 | 7.61 (5.25,11.05) | 2.91 (2.01,4.23) | 7.54 (5.19,10.94) |
| lenvatinib | small intestinal perforation | 14 | 12.70 (7.49,21.54) | 3.65 (2.15,6.18) | 12.51 (7.38,21.22) |
| lenvatinib | rectal perforation | 3 | 10.07 (3.22,31.43) | 3.32 (1.06,10.35) | 9.95 (3.19,31.08) |
| lenvatinib | oesophageal perforation | 15 | 22.88 (13.7,38.24) | 4.48 (2.68,7.48) | 22.27 (13.33,37.21) |
| lenvatinib | lower gastrointestinal perforation | 4 | 109.17 (38.46,309.91) | 6.59 (2.32,18.71) | 96.42 (33.97,273.73) |
| lenvatinib | large intestine perforation | 34 | 6.12 (4.37,8.58) | 2.6 (1.86,3.65) | 6.07 (4.33,8.51) |
| lenvatinib | intestinal perforation | 56 | 6.17 (4.74,8.03) | 2.61 (2.01,3.4) | 6.12 (4.70,7.96) |
| lenvatinib | gastrointestinal perforation | 40 | 11.84 (8.66,16.18) | 3.54 (2.59,4.84) | 11.66 (8.53,15.93) |
| lenvatinib | gastric perforation | 12 | 5.71 (3.24,10.08) | 2.5 (1.42,4.42) | 5.68 (3.22,10.01) |
| lenvatinib | duodenal perforation | 12 | 16.09 (9.08,28.49) | 3.98 (2.25,7.05) | 15.79 (8.91,27.96) |
| lenvatinib | diverticular perforation | 5 | 2.69 (1.12,6.46) | 1.42 (0.59,3.42) | 2.68 (1.11,6.45) |
| nintedanib | lower gastrointestinal perforation | 3 | 56.79 (17.36,185.78) | 5.7 (1.74,18.64) | 51.86 (15.85,169.66) |
| nintedanib | large intestine perforation | 17 | 2.18 (1.36,3.52) | 1.12 (0.70,1.81) | 2.18 (1.35,3.51) |
| nintedanib | intestinal perforation | 28 | 2.2 (1.52,3.19) | 1.13 (0.78,1.64) | 2.19 (1.51,3.18) |
| nintedanib | gastrointestinal perforation | 17 | 3.57 (2.22,5.75) | 1.83 (1.14,2.95) | 3.55 (2.21,5.73) |
| nintedanib | gastric perforation | 8 | 2.72 (1.36,5.45) | 1.44 (0.72,2.88) | 2.71 (1.35,5.44) |
| nintedanib | diverticular perforation | 7 | 2.7 (1.28,5.67) | 1.43 (0.68,3.00) | 2.69 (1.28,5.65) |
| nivolumab | small intestinal perforation | 17 | 3.65 (2.26,5.89) | 1.85 (1.14,2.98) | 3.6 (2.23,5.81) |
| nivolumab | oesophageal perforation | 9 | 3.20 (1.66,6.18) | 1.66 (0.86,3.21) | 3.16 (1.64,6.11) |
| nivolumab | large intestine perforation | 54 | 2.3 (1.76,3.01) | 1.19 (0.91,1.56) | 2.28 (1.75,2.99) |
| nivolumab | jejunal perforation | 6 | 7.43 (3.29,16.79) | 2.85 (1.26,6.43) | 7.19 (3.18,16.25) |
| nivolumab | intestinal perforation | 74 | 1.92 (1.53,2.42) | 0.94 (0.74,1.18) | 1.91 (1.52,2.41) |
| nivolumab | gastrointestinal perforation | 51 | 3.57 (2.70,4.71) | 1.82 (1.38,2.40) | 3.52 (2.67,4.64) |
| nivolumab | duodenal perforation | 11 | 3.47 (1.91,6.30) | 1.78 (0.98,3.23) | 3.43 (1.89,6.22) |
| panitumumab | small intestinal perforation | 3 | 4.02 (1.29,12.48) | 2 (0.64,6.22) | 4.01 (1.29,12.45) |
| panitumumab | rectal perforation | 4 | 20.15 (7.50,54.14) | 4.31 (1.60,11.58) | 19.84 (7.38,53.29) |
| panitumumab | large intestine perforation | 12 | 3.21 (1.82,5.66) | 1.68 (0.95,2.96) | 3.2 (1.82,5.65) |
| panitumumab | intestinal perforation | 17 | 2.78 (1.73,4.48) | 1.47 (0.91,2.37) | 2.78 (1.72,4.47) |
| panitumumab | ileal perforation | 4 | 11.18 (4.18,29.93) | 3.47 (1.30,9.29) | 11.09 (4.14,29.67) |
| panitumumab | gastrointestinal perforation | 12 | 5.25 (2.98,9.26) | 2.39 (1.35,4.21) | 5.23 (2.96,9.22) |
| pazopanib | small intestinal perforation | 8 | 5.59 (2.79,11.21) | 2.47 (1.23,4.96) | 5.55 (2.77,11.13) |
| pazopanib | rectal perforation | 3 | 7.81 (2.50,24.37) | 2.95 (0.94,9.21) | 7.72 (2.47,24.11) |
| pazopanib | large intestine perforation | 20 | 2.78 (1.79,4.32) | 1.47 (0.95,2.28) | 2.77 (1.79,4.30) |
| pazopanib | jejunal perforation | 3 | 11.98 (3.82,37.54) | 3.56 (1.14,11.15) | 11.77 (3.76,36.90) |
| pazopanib | intestinal perforation | 51 | 4.35 (3.30,5.73) | 2.11 (1.60,2.78) | 4.32 (3.28,5.69) |
| pazopanib | ileal perforation | 3 | 4.34 (1.39,13.50) | 2.11 (0.68,6.57) | 4.32 (1.39,13.43) |
| pazopanib | gastrointestinal perforation | 23 | 5.24 (3.47,7.90) | 2.38 (1.58,3.59) | 5.2 (3.45,7.84) |
| pembrolizumab | small intestinal perforation | 20 | 6.63 (4.26,10.33) | 2.7 (1.73,4.21) | 6.51 (4.18,10.14) |
| pembrolizumab | large intestine perforation | 26 | 1.70 (1.15,2.49) | 0.76 (0.52,1.11) | 1.69 (1.15,2.49) |
| pembrolizumab | jejunal perforation | 6 | 11.44 (5.06,25.86) | 3.47 (1.53,7.83) | 11.05 (4.89,24.98) |
| pembrolizumab | duodenal perforation | 6 | 2.89 (1.29,6.46) | 1.52 (0.68,3.40) | 2.87 (1.29,6.42) |
| regorafenib | large intestine perforation | 11 | 2.64 (1.46,4.76) | 1.39 (0.77,2.52) | 2.63 (1.45,4.75) |
| regorafenib | gastrointestinal perforation | 19 | 7.46 (4.75,11.72) | 2.89 (1.84,4.54) | 7.41 (4.72,11.64) |
| regorafenib | gastric perforation | 5 | 3.17 (1.32,7.63) | 1.66 (0.69,4.00) | 3.16 (1.32,7.61) |
| regorafenib | duodenal perforation | 4 | 7.08 (2.65,18.94) | 2.82 (1.05,7.53) | 7.04 (2.63,18.83) |
| rituximab | small intestinal perforation | 16 | 2.29 (1.40,3.76) | 1.18 (0.72,1.94) | 2.27 (1.38,3.72) |
| rituximab | intestinal perforation | 98 | 1.71 (1.40,2.09) | 0.76 (0.63,0.93) | 1.7 (1.39,2.07) |
| rituximab | gastric perforation | 24 | 1.81 (1.21,2.71) | 0.85 (0.57,1.27) | 1.8 (1.20,2.69) |
| rituximab | duodenal ulcer perforation | 81 | 6.42 (5.14,8.03) | 2.62 (2.10,3.28) | 6.16 (4.93,7.70) |
| rituximab | diverticular perforation | 29 | 2.5 (1.73,3.60) | 1.3 (0.90,1.88) | 2.47 (1.71,3.56) |
| sorafenib | large intestine perforation | 31 | 3.82 (2.68,5.44) | 1.92 (1.35,2.74) | 3.8 (2.67,5.40) |
| sorafenib | intestinal perforation | 25 | 1.88 (1.27,2.78) | 0.9 (0.61,1.34) | 1.87 (1.26,2.77) |
| sorafenib | gastrointestinal perforation | 30 | 6.05 (4.22,8.68) | 2.58 (1.80,3.70) | 5.99 (4.18,8.59) |
| sorafenib | duodenal perforation | 4 | 3.62 (1.36,9.69) | 1.85 (0.69,4.95) | 3.61 (1.35,9.64) |
| sorafenib | diverticular perforation | 9 | 3.32 (1.72,6.40) | 1.73 (0.90,3.32) | 3.31 (1.72,6.37) |
| sunitinib | rectal perforation | 4 | 4.43 (1.65,11.90) | 2.13 (0.79,5.72) | 4.38 (1.63,11.75) |
| sunitinib | oesophageal perforation | 5 | 2.46 (1.02,5.94) | 0.54 (0.17,3.11) | 2.45 (1.01,5.90) |
| sunitinib | intestinal perforation | 72 | 2.61 (2.07,3.29) | 1.37 (1.09,2.73) | 2.59 (2.06,3.27) |
| sunitinib | gastrointestinal perforation | 24 | 2.32 (1.55,3.46) | 1.21 (0.81,2.80) | 2.31 (1.54,3.45) |
| sunitinib | gastric perforation | 13 | 2.03 (1.18,3.51) | 1.02 (0.59,1.76) | 2.03 (1.17,3.50) |
| sunitinib | duodenal perforation | 7 | 3.06 (1.45,6.44) | 1.6 (0.76,3.37) | 3.04 (1.44,6.39) |
| sunitinib | diverticular perforation | 17 | 3.03 (1.88,4.88) | 1.59 (0.98,2.56) | 3.00 (1.86,4.84) |
| temsirolimus | small intestinal perforation | 3 | 12.92 (4.16,40.15) | 3.69 (1.19,11.46) | 12.87 (4.14,40.00) |
| temsirolimus | large intestine perforation | 7 | 6.02 (2.87,12.64) | 2.59 (1.23,5.43) | 6 (2.86,12.61) |
| temsirolimus | intestinal perforation | 10 | 5.26 (2.83,9.79) | 2.39 (1.28,4.45) | 5.24 (2.82,9.76) |
| trametinib | intestinal perforation | 15 | 4.06 (2.45,6.74) | 2.02 (1.22,3.35) | 4.05 (2.44,6.72) |
| vandetanib | large intestine perforation | 4 | 7.85 (2.94,20.95) | 2.97 (1.11,7.92) | 7.82 (2.93,20.88) |
| vandetanib | intestinal perforation | 4 | 4.8 (1.80,12.80) | 2.26 (0.85,6.03) | 4.78 (1.79,12.77) |
| vandetanib | diverticular perforation | 3 | 17.67 (5.69,54.92) | 4.14 (1.33,12.86) | 17.61 (5.67,54.72) |
| zanubrutinib | intestinal perforation | 3 | 18.21 (5.84,56.80) | 4.17 (1.34,13.01) | 18.04 (5.79,56.26) |

N, number of adverse event reports.
